# Sociocultural beliefs and palliative care needs among older adults in low- and middle-income countries: A narrative review

**DOI:** 10.4102/phcfm.v18i1.5434

**Published:** 2026-08-04

**Authors:** Hussaini Y. Magaji, Felix Archibong, Monica N. Adekoya, Ekanem A. Edet, Abdulgafar L. Olawumi, Attahiru Muhammad, Muhammad H. Dauda

**Affiliations:** 1 Department of Family Medicine, Faculty of Clinical Science, Rasheed Shekoni Federal University Teaching Hospital, Dutse, Nigeria; 2 Department of Family Medicine, Faculty of Clinical Science, University of Calabar, Calabar, Nigeria; 3 Department of Child Dental Health, Faculty of Dentistry, University of Calabar, Calabar, Nigeria; 4 Department of Family Medicine, Faculty of Clinical Science, University of Alberta, Alberta, Canada; 5 Department of Family Medicine, Faculty of Clinical Science, Aminu Kano Teaching Hospital, Kano, Nigeria; 6 Department of Family Medicine, Faculty of Clinical Science, Abubakar Tafawa Balewa University Teaching Hospital, Bauchi, Nigeria

**Keywords:** palliative care, older adults, sociocultural beliefs, ageing, low-income and middle-income countries

## Abstract

Palliative care is an integral component of comprehensive primary health care and is particularly relevant for older adults living with chronic and progressive illnesses. In low- and middle-income countries (LMICs), including many African settings, access to palliative care remains limited despite substantial need. Sociocultural beliefs shape perceptions of ageing, illness, suffering and death, and influence how palliative care needs are recognised and addressed within primary care and family medicine contexts. To synthesise existing evidence on how sociocultural beliefs influence palliative care needs and utilisation among older adults in LMICs, with implications for primary care and family practice. A narrative literature review was conducted using published qualitative, quantitative and mixed-methods studies identified through searches of major electronic databases and relevant grey literature. Evidence was synthesised thematically, focusing on sociocultural influences relevant to primary care settings. Key themes identified included cultural constructions of ageing and suffering, family-centred decision-making, religious and spiritual interpretations of illness, and misconceptions surrounding palliative care. These factors influence symptom reporting, care-seeking behaviour, referral patterns and utilisation of palliative care services at the primary care level. Sociocultural beliefs are fundamental to understanding palliative care needs among older adults in LMICs. To improve access and quality of care, culturally sensitive palliative care models that involve families, recognise spiritual concerns and dispel myths are crucial. More empirical research from under-represented LMIC regions is required to inform contextually appropriate policy and practice.

## Introduction

The world’s population is getting older faster, with low- and middle-income countries (LMICs) experiencing the highest growth.^1^ People who live longer are more susceptible to multimorbidity, frailty, chronic non-communicable diseases and progressive functional deterioration. Distressing symptoms like pain, dyspnoea, exhaustion, sadness and anxiety often accompany these illnesses and significantly lower quality of life. Palliative care is an essential part of comprehensive treatment for older adults since it has been demonstrated to enhance symptom control, psychological well-being and care coordination for those with serious disease.^2^ Globally, an estimated 40% – 60% of people who died experiencing serious health-related suffering would have benefited from palliative care, but coverage is still severely inadequate.^3^ Access to palliative care services is especially restricted in LMICs, where the majority of the world’s elderly population lives. While structural barriers like lack of trained health workers, shortage of opioids and poor health systems are well-documented, there is growing recognition that sociocultural factors also play a significant role in determining palliative care need, demand and utilisation.^4^

Recent narrative and integrative reviews have highlighted the importance of cultural, social and spiritual contexts in shaping end-of-life experiences and engagement with palliative care, particularly in resource-limited and culturally diverse settings.^5,6,7^ These reviews underscore attitudes toward ageing. Suffering and death are deeply embedded within cultural value systems and family structures, with important implications for palliative care delivery. Sociocultural beliefs influence how older people and their families perceive suffering, understand illness trajectories, give meaning to ageing and make decisions about end-of-life care. Ageing may be seen in many LMIC contexts as a normal process linked to inevitable deterioration, which could normalise suffering and deter people from seeking help from a palliative care provider. Palliative care is frequently misinterpreted as being equivalent to impending death or giving up on curative therapy, which causes stigma and delays in referrals.^8^ Despite the importance of these issues, the sociocultural dimensions of palliative care among older adults in LMICs remain underexplored and fragmented across disciplines.

This narrative review aims to synthesise and critically examine the existing literature on sociocultural beliefs and palliative care needs among older adults in LMICs. By integrating evidence across diverse settings, the review seeks to elucidate how culture, family structures, religion and spirituality shape experiences of serious illness and engagement with palliative care, and to identify gaps that warrant further investigation.

## Conceptual framing

### Palliative care and ageing

Palliative care is an approach that improves the quality of life for patients and their families facing life-threatening illness by preventing and relieving suffering, early identifying and treating pain and other problems, whether physical, psychosocial or spiritual.^3^ For older adults, palliative care is particularly relevant because of the cumulative burden of chronic illness, functional impairment and social vulnerability. Ageing is not merely a biological process but is deeply embedded within social and cultural contexts. Cultural norms shape expectations regarding independence, family responsibility and acceptable expressions of pain or distress. These norms, in turn, influence when palliative care needs are recognised and whether such care is perceived as appropriate.^8^

### Sociocultural beliefs and health-seeking behaviour

Sociocultural beliefs encompass shared values, norms, traditions and worldviews that influence how individuals interpret health and illness. Anthropological and sociological frameworks emphasise that illness experiences are socially constructed and mediated by cultural meaning systems.^9^ In LMIC settings, pluralistic health systems in which biomedical care coexists with traditional and faith-based healing further shape care-seeking pathways. Religion and spirituality are particularly salient in many LMICs, providing explanatory models for illness, suffering and influencing coping strategies and end-of-life preferences. Family structures and collective decision-making norms also play a central role, often positioning relatives as key gatekeepers in care decisions.^4^

## Methods: Narrative review approach

A narrative literature review approach was employed to synthesise evidence on sociocultural beliefs and palliative care needs among older adults in LMICs. Relevant literature was identified through searches of major electronic databases, including PubMed, Index to Nursing and Allied Health Literature (CINAHL), Scopus and Google Scholar, using combinations of keywords related to palliative care, ageing, sociocultural beliefs, religion, spirituality, family decision-making and LMICs. Grey literature from international organisations and regional palliative care networks was also consulted. The search yielded a diverse but heterogeneous body of mainly small, context-specific studies from LMICs, with variable methodological quality that may limit generalisability.

Empirical studies employing qualitative, quantitative or mixed-methods designs were purposively selected based on relevance to the review objectives. The literature was read iteratively and analysed thematically, with attention to recurring patterns, contextual influences and points of convergence or divergence across studies. Rather than providing an exhaustive catalogue of studies, the review offers a critical synthesis of key themes that characterise the sociocultural dimensions of palliative care for older adults in LMICs. Although the search strategy’s use of several databases and appropriate keywords increased the volume of literature found, it lacked the rigour of a complete systematic approach, which would have included thorough grey literature searching and consistent use of restricted terminology. As a result, a few relevant research studies might have been overlooked, and the results should be evaluated cautiously.

## Thematic synthesis

### Cultural constructions of ageing, illness and suffering

Across many LMIC settings, ageing is often conceptualised as a natural life stage accompanied by physical decline and increasing dependency. Such perceptions may lead older adults and their families to view pain, weakness and functional loss as inevitable rather than as symptoms warranting medical attention. Several studies report that this normalisation of suffering contributes to delayed presentation and under-recognition of palliative care needs.^10,11^ Cultural norms that value endurance, stoicism and acceptance may further discourage older adults from expressing distress. In some contexts, articulating pain or emotional suffering is perceived as a sign of weakness or a burden on family members. These beliefs can obscure the true extent of symptom burden and limit opportunities for timely palliative interventions.^7,12^

### Family-centred decision-making and care dynamics

Family plays a central role in the care of older adults in LMICs, often serving as the primary source of physical, emotional and financial support. Decision-making around serious illness and end-of-life care is frequently collective rather than individual, with family members acting as intermediaries between patients and healthcare providers.^5,13^ While family involvement can provide protection and support, it may also complicate palliative care engagement. Relatives may withhold information about prognosis to protect the older person from distress, or insist on pursuing aggressive treatments because of moral obligations or social expectations. Such dynamics can delay palliative care referral and limit patient autonomy.^6,14^

### Religion, spirituality and meaning-making

Religion and spirituality are deeply intertwined with health and illness experiences in many LMICs. Older adults often draw on religious beliefs to interpret suffering, viewing illness as a test of faith, divine will or an opportunity for spiritual growth.^15,16^ These beliefs can foster acceptance and resilience, but may also lead to ambivalence toward palliative care if suffering is perceived as spiritually meaningful. Faith-based coping strategies, including prayer and consultation with religious leaders, are commonly used alongside or in place of biomedical care. In some cases, strong reliance on spiritual healing may delay engagement with palliative services, particularly when such services are perceived as incompatible with hope or faith.^17,18^

### Misconceptions and stigma surrounding palliative care

A recurrent theme in the literature is widespread misunderstanding of palliative care.^18,19,20^ Many older adults, families and even healthcare providers equate palliative care with imminent death or the cessation of curative treatment. This misconception contributes to fear, stigma and late referral, often limiting palliative care to the final days or weeks of life.^14,21^ In LMIC contexts, where awareness of palliative care remains low, these misconceptions are compounded by limited-service availability and weak integration into routine care. As a result, older adults with significant unmet needs may never access palliative support.^7,22^

## Discussion

The recognition of palliative care needs and the use of palliative services by older persons in LMICs are significantly influenced by sociocultural views, as this narrative review demonstrates. The review shows how attitudes of ageing, suffering, family responsibilities, religion and palliative care itself have a significant impact on care-seeking activity and referral timing, based on findings from various cultural and health system contexts.

In many LMIC contexts, ageing is generally seen as a normal stage of inevitable decline, with dependency, pain and functional loss being normalised rather than medicalised. Qualitative studies from Asia and Africa have consistently shown that these cultural constructions lead to delayed presentation to health facilities and under-recognition of palliative care needs.^10,11^ These results are consistent with anthropological viewpoints that highlight how sickness is socially constructed and how cultural norms influence how people communicate their symptoms and seek assistance.^12^

Palliative care participation has been found to be significantly influenced by family-centred decision-making. Families serve as older individuals’ primary caregivers and moral decision-makers in many LMIC settings, frequently putting group values ahead of personal autonomy. Palliative care referrals may be delayed when families seek curative treatments or suppress prognostic information to protect the patient, even though such arrangements can offer significant emotional and practical support.^5,6^ Similar patterns have been noted in studies conducted in Nigeria, where end-of-life decisions are heavily influenced by social expectations and familial obligations.^8^

The main frameworks that older individuals use to understand illness and suffering were consistently found to be religion and spirituality. Faith-based explanations, such as seeing illness as a test of faith or a manifestation of divine will, can promote psychological resilience and acceptance, but if pain is given spiritual significance, they may also cause ambivalence regarding palliative care.^15,16^ Strong dependence on prayer and religious healing may postpone using biomedical and palliative therapies, according to studies from South Asia and sub-Saharan Africa, especially if palliative care is seen as incompatible with hope.^17,18^

Palliative care misconceptions are a significant and enduring impediment. This narrative review confirms the common misconception that palliative care is synonymous with impending death or treatment abandonment. This misconception has been documented in both high-income and low-income settings, but it is especially noticeable when services are not well integrated into routine care.^14,21^ Palliative care’s potential advantages are undermined in LMICs as a result of low public knowledge and late-stage service availability, which reinforces stigma and limits it to the last stage of disease.^3,7^

Crucially, disparities in access to palliative care are exacerbated by the interplay between structural health system limitations and social views. Cultural norms combine with a lack of workers, restricted access to opioids and inadequate referral processes to result in care that is postponed or skipped. This emphasises the need for palliative care strategies that are culturally sensitive, integrated into primary care, and in line with regional societal realities.^4,23^ Overall, the findings of this review reinforce calls for a more holistic approach to palliative care development in LMICs, one that integrates cultural competence, family engagement and spiritual care alongside system-level strengthening. Addressing sociocultural barriers through community education, provider training and culturally sensitive communication is essential to improving timely access and quality of palliative care for older adults.

## Gaps and future research directions

Despite growing interest in palliative care in LMICs, empirical evidence on sociocultural beliefs among older adults remains limited and unevenly distributed geographically. There is a particular paucity of data from sub-Saharan Africa, South Asia and fragile health system contexts, with much of the existing literature concentrated in a small number of countries. Many available studies are cross-sectional or qualitative, limiting causal inference and generalisability, and few explicitly disaggregate findings by age within older populations. Future research should employ longitudinal and mixed-methods designs to explore how sociocultural beliefs evolve and interact with health system factors and should prioritise under-represented regions and community-based settings.

### Limitations of the review

As a narrative synthesis, the review did not employ a systematic or scoping review methodology. It therefore may not have captured all relevant studies on sociocultural beliefs and palliative care in LMICs. Study selection was purposive and guided by relevance rather than exhaustive coverage, introducing the possibility of selection bias. In addition, the heterogeneity of study designs and contexts limited direct comparison across settings. Nevertheless, the narrative approach allowed for in-depth conceptual integration of diverse evidence, which is appropriate for exploring complex sociocultural phenomena.

## Conclusion

Sociocultural beliefs play a central role in shaping palliative care needs and utilisation among older adults in LMICs. Cultural constructions of ageing and suffering, family-centred decision-making, religious and spiritual interpretations of illness, and persistent misconceptions about palliative care all influence how and when older adults engage with care. Addressing these factors is essential for developing equitable and effective palliative care services that respond to the lived realities of ageing populations in resource-limited settings.
